# In Search for a Pathogenesis of Major Depression and Suicide—A Joint Investigation of Dopamine and Fiber Tract Anatomy Focusing on the Human Ventral Mesencephalic Tegmentum: Description of a Workflow

**DOI:** 10.3390/brainsci14070723

**Published:** 2024-07-18

**Authors:** Jana M. Zielinski, Marco Reisert, Bastian E. A. Sajonz, Shi Jia Teo, Annette Thierauf-Emberger, Johanna Wessolleck, Maximilian Frosch, Björn Spittau, Jochen Leupold, Máté D. Döbrössy, Volker A. Coenen

**Affiliations:** 1 Department of Stereotactic and Functional Neurosurgery, Medical Center of Freiburg University, Breisacher Straße 64, 79106 Freiburg i.Br., Germany; 2 Medical Faculty of University of Freiburg, 79106 Freiburg, Germany; 3 Department of Diagnostic and Interventional Radiology, Medical Physics, Medical Center—University of Freiburg, 79106 Freiburg, Germany; 4 Institute of Forensic Medicine, Medical Center of Freiburg University, 79104 Freiburg, Germany; 5 Laboratory of Stereotaxy and Interventional Neurosciences, Department of Stereotactic and Functional, Neurosurgery, Medical Center of Freiburg University, 79106 Freiburg, Germany; 6 Institute of Neuropathology, Medical Center of Freiburg University, 79106 Freiburg, Germany; 7 Medical School OWL, Anatomy and Cell Biology, Bielefeld University, 33501 Bielefeld, Germany; 8 Institute for Anatomy and Cell Biology, Department of Molecular Embryologie, Faculty of Medicine, Freiburg University, 79104 Freiburg, Germany; 9 Faculty of Biology, University of Freiburg, 79104 Freiburg, Germany; 10 Center for Deep Brain Stimulation, Medical Center of Freiburg University, 79106 Freiburg, Germany; 11 Center for Basics in Neuromodulation, Medical Faculty of Freiburg University, 79106 Freiburg, Germany

**Keywords:** connectivity, dopamine, major depressive disorder, midbrain, suicide, subnuclei, ventral tegmental area

## Abstract

Major depressive disorder (MDD) is prevalent with a high subjective and socio-economic burden. Despite the effectiveness of classical treatment methods, 20–30% of patients stay treatment-resistant. Deep Brain Stimulation of the superolateral branch of the medial forebrain bundle is emerging as a clinical treatment. The stimulation region (ventral tegmental area, VTA), supported by experimental data, points to the role of dopaminergic (DA) transmission in disease pathology. This work sets out to develop a workflow that will allow the performance of analyses on midbrain DA-ergic neurons and projections in subjects who have committed suicide. Human midbrains were retrieved during autopsy, formalin-fixed, and scanned in a Bruker MRI scanner (7T). Sections were sliced, stained for tyrosine hydroxylase (TH), digitized, and integrated into the Montreal Neurological Institute (MNI) brain space together with a high-resolution fiber tract atlas. Subnuclei of the VTA region were identified. TH-positive neurons and fibers were semi-quantitatively evaluated. The study established a rigorous protocol allowing for parallel histological assessments and fiber tractographic analysis in a common space. Semi-quantitative readings are feasible and allow the detection of cell loss in VTA subnuclei. This work describes the intricate workflow and first results of an investigation of DA anatomy in VTA subnuclei in a growing naturalistic database.

## 1. Introduction

Major depressive disorder (MDD) is a debilitating disease affecting approximately 280 million patients worldwide (Institute of Health Metrics and Evaluation, Global Health Data Exchange (GHDx), https://vizhub.healthdata.org/gbd-results/, accessed 4 March 2023). Around 800,000 suicides per year worldwide are related to MDD. Therapy options exist, like psychotherapy and antidepressants, and are most efficient when used in combination [1]. MDD is viewed as a network disorder where distinct sub-networks or the interaction between them are disturbed [2]. According to the “monoamine hypothesis”, depression symptoms arise as a result of a decrease of available monoamines, such as dopamine (DA), noradrenaline, or serotonin, in the synaptic cleft in CNS areas associated with motivation and emotion regulation [3]. Despite numerous other hypotheses concerning the etiology of MDD [4], the “monoamine hypothesis” is still relevant today, although more so as an explanation for deficient network interactions [5] rather than as a primary mechanism of its own, as previously perceived. Antidepressant therapies are sufficient in most patients [6]. Yet, there is a significant proportion of patients in whom conventional therapies, including electroconvulsive therapy [7], are ineffective. In these even more severe treatment-resistant disease states, another promising therapy option is Deep Brain Stimulation (DBS). Numerous targets have been regarded in a significant number of single or single-center trials [8], among them the subgenual cingulate gyrus, the ventral capsule ventral striatum, and the superolateral branch of the medial forebrain bundle (slMFB) [9]. DBS of the latter shows promising results in outcome and sustained improvement of patients who suffer from MDD [9,10,11,12], and it has been speculated that DBS of the VTA (targeting the slMFB) antidromically affects cortical fields reached by DA projections [13]. Clinically, these patients respond rapidly with an appetitive motivation response reminiscent of a DA mechanism [14,15]. The ascending midbrain mesocorticolimbic dopamine projection (A10 DA system), considered to be one of several hard-wired primary affective systems, originates in the VTA and projects via the mfb (lower case used in rodents to distinguish it from the primate pathway) to the NAC and prefrontal cortex (PFC) [16]. The projection pathway, confluent with the SEEKING system in the affective neuroscience literature, serves as the neural foundation for positive emotions and euphoric behaviors that facilitate exploration/motivation and regulate appetitive learning [17]. Among other mechanisms, DA transmission is responsible for flexible decision-making. Key symptoms in depression, such as anhedonia and reduced appetitive motivation levels, can—at least partially—be explained by dysfunctional DA transmission through these projection pathways [18]. In conjunction, the DA transmission patterns show a different DBS response in depression-like phenotypes than in healthy control animals [19], indicating the special importance of this transmitter [18,20,21,22,23,24]. DA receptor polymorphism plays an important role in the explanation of depression and suicide genesis. However, according to a recent review of the topic, the role of the distinct DA receptors (D1-D5) in the genesis of depression is not entirely clear [25]. The D2 receptor is the most common receptor responsible for the action of antipsychotic drugs. Ebert et al. reported that selective Serotonin reuptake inhibitor (SSRI) responsiveness is related to striatal D2 receptor binding [26]. The D2 receptor subtype is coded via the DRD2 gene. An association with adolescent depression was found with the A241G polymorphism [27]. Moreover, DRD2 receptor gene polymorphism might be involved in suicide behavior [28].

Sunlight exposure and MDD: It is not uncommon to hear lay people talk about a connection between sunlight exposure and symptoms of depression. Scientifically, there is a debate around this fact [29,30,31,32], although it is potentially fair to say that there is a role of sunlight exposure in depression severity. If this holds true for MDD is not clear. Auman and coworkers have found an interesting correlation between hypothalamic and midbrain DA neuron counts and duration of exposure to sunlight [33,34]. Since the amount of DA cells in the substantia nigra is related to specific behavior and environmental factors [34], we sought to look at the factor of sunlight exposure in our work, too.

If there is such relevance for DA transmission, could a mere loss or the alteration of the connectivity of DA neurons in the VTA—and its sub-nuclei—contribute to MDD pathophysiology, just as a “depressed” phenotype in animals is associated with DA neuronal function loss? To our knowledge this question has not been addressed in a broader scope and with a detailed look at VTA anatomy. In this proof-of-concept study, we investigated with integration of histology, transmitter staining (with semi-quantitative analysis of DA), and parallel presentation of high-resolution fiber tractography in a normalized space the cellular VTA DA alteration in a small number of subjects who had committed suicide as a potential indicator for MDD. We hypothesize that there might be a loss of DA neurons or other cellular structures (interneurons) in sub-parts of the VTA related to MDD and suicide. This paper sets out to describe the intricate analysis pathway necessary for such research. Regarding DA transmission we present some very preliminary pilot data exemplifying our principle approach.

## 2. Material and Methods

### 2.1. Ethics

Most of the subjects included at the time of their death were presumed to have committed suicide. For this proof-of-concept study, fresh midbrain specimens were acquired during an autopsy at the Department of Forensic Pathology (Freiburg University). Autopsies were based on the German criminal procedure code, and samples represent material that is gained, when possible, in every autopsy case. Sampling is in accordance with the statement and the first supplement of the central ethics commission of the German Medical Association [35,36]. Therefore, obtaining individual consent was not deemed necessary for this proof-of-concept study. Before publication, the local IRB was consulted (IRB vote under no. 24-1276, 18 June 2024). The Ethics Committee has no objections to the publication of these exploratory investigation results. The Ethics Committee assumes that the data are anonymized and that only anonymized data will be published. The single donated brain (for the creation of the fiber atlas) was obtained in collaboration with the University of Freiburg, Department of Anatomy, and its histological assessment was carried out with the approval of the local ethical committee (no. 203/11). Details can be found in our previous work [13].

### 2.2. Workflow

**Figure 1:**
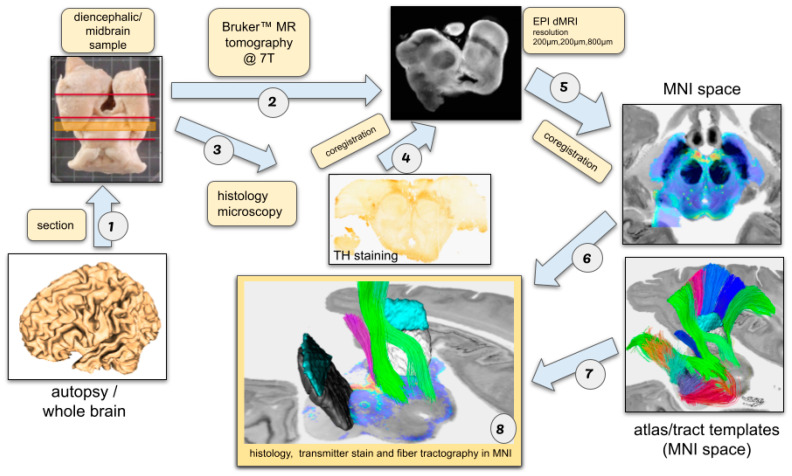
Workflow. After sectioning the midbrain out of the whole brain (1), the midbrain was imaged using 7T small animal scanner (Bruker Biospec, Boston, MA, USA) utilizing an EPI diffusion-weighted MRI sequence (2). After MRI acquisition, the sample underwent the proposed histological staining protocol (3). Slice sections obtained from histology were semi-manually coregistered to the dMRI tomographic dataset in a deformable manner (4) using NORA (www.nora-imaging.org, accessed on 1 May 2024). To relate the histological sections with the common MNI template space, the dMRI dataset was registered to the MNI space using the ANTs toolbox (5). For this coregistration step, fiber density maps generated from a tractographic analysis were used. Finally, the transforms from (4–5) were combined to bring the histological slices directly to the MNI space, where they were overlaid with tract atlas information (5–7). The final result is a combined digital rendition of histology, transmitter stain and fiber tractography (8).

A principle workflow of the utilized techniques is shown in Figure 1.

#### 2.2.1. Midbrain Samples

Autopsies were performed in the Department of Forensic Medicine, Freiburg. During autopsy, the whole brain was removed from the cranial cavity through a calvarial opening by standard procedure. Strategic cuts were made to preserve a specimen containing the midbrain and part of the diencephalic—mesencephalic junction. At first, a protocol for further sequential processing was established. After adjusting several parameters in the ongoing experiment, due to the limited availability of fitting postmortem human tissue, the study was then performed on the 3 samples described below (Table 1, S3–S5). The samples were stored in a formalin-buffered solution.

#### 2.2.2. MRI-Measurement, Transfer into a Common Space, and Tractographic Analysis

**Figure 2:**
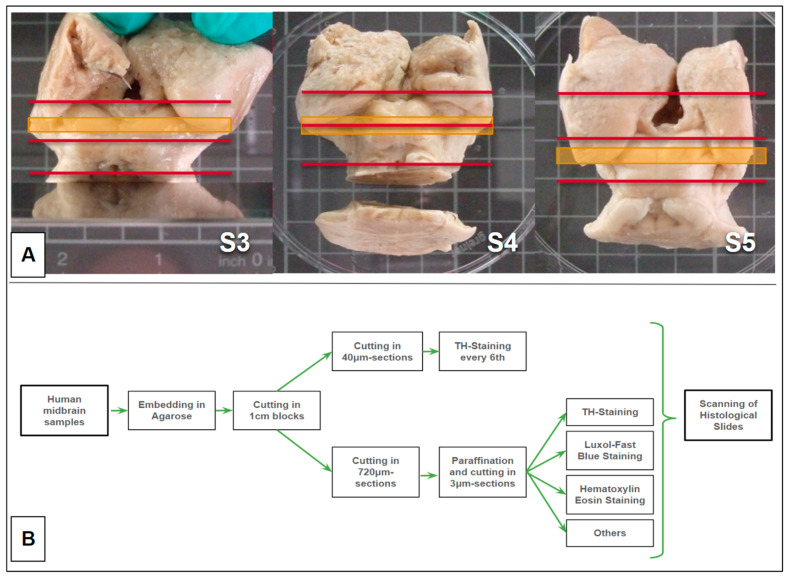
(**A**) Typical autopsy samples. Specimens 3-5 (S3-S5).Orange lines indicate the evaluated sample slice. Red lines indicate the range of sampling as indicated in (**B**). (**B**) workflow of histological staining.

The midbrain was separated from the entire brain (Figure 1, step 1 and Figure 2A) and then captured using a 7T Bruker Biospec 70/20 scanner, employing a diffusion-weighted MRI technique (Figure 1, step 2). Postmortem brainstem scans were conducted with a 200 mm bore, using a 1H volume coil from RAPID Biomedical. Diffusion data were captured in three thick slabs using a 3D spin-echo DTI sequence, with a TE = 30 ms, TR = 310 ms, 0.208 × 0.208 × 0.833 mm. All slabs were imaged using one b0 image and 50 diffusion directions with a b-value of 8000 s/mm^2^. Post MRI, the specimen was subjected to the outlined histological staining process (Figure 1, step 3). Subsequently, the histological sections were aligned with the dMRI tomographic data through a semi-manual, flexible coregistration process (Figure 1, step 4) utilizing NORA (www.nora-imaging.org, accessed on 1 May 2024) Based on a set of manually chosen key points, which were based on anatomical landmarks, a warp field was computed to align the sections and the dMRI tomography. To eventually correlate these histological sections with the standard MNI template, the dMRI data were aligned to the MNI space [37] using the ANTs software (https://stnava.github.io/ANTs/, accessed on 1 May 2024). To obtain a robust and reliable contrast for coregistration of the individual dMRI data and our MNI template, we relied on streamline densities obtained from a global tracking approach [38], i.e., the alignment is based on the directional information obtained from the DTI measurement. The parameters used were adopted from the suggestions proposed in [13]. Prior to tractography, the dMRI data were denoised using random-matrix theory [39] and upsampled to a resolution of 0.208 × 0.208 × 0.370 mm by an edge-preserving upsampling scheme [40,41].

The final step involved merging the transformation processes from (Figure 1, steps 4 and 5), enabling the positioning of the histological sections directly into the MNI space, where they were integrated with the tract atlas [13] data (Figure 1, steps 5–7).

#### 2.2.3. Preparation

Preparation steps are shown in Figure 2. The tissue was embedded in agarose, then cut into 1 cm slices parallel to the ACPC plane (under MRI guidance), and stored in 30% sucrose until it sank. Afterward, referring to [42], the slices containing the VTA were selected and cut on a cryogenic microtome in the following chronological order: Starting with six times 40 µm sections stored in antifreeze, this step was followed by cutting a 720 µm part stored in 5% PFA, then six times 40 µm sections and one time 720 µm again until finishing the block.

#### 2.2.4. TH Staining

The last of every 40 µm section was chosen as representative and further stained using the Tyrosine Hydroxylase-antibody (TH-antibody). The 720 µm slices were dehydrated and embedded in paraffin for further cutting into 3 µm sections. After mounting the 40 µm-sections on chrome-gelatine coated double-sized glass slides, they were first washed with DAKO wash solution in a ratio of 1:10 and then incubated in a quenching solution consisting of 50 mL H_2_O_2_ and 50 mL Methanol in 400 mL phosphate-buffered saline (PBS) at pH 7.4. Afterward, the sections were washed again and transferred into a blocking solution consisting of 15 g Bovine Serum Albumin (SIGMA #SLCH0396, Merck SA, Darmstadt, Germany) and 1.5 mL Triton-X (SIGMA #SLCD3237) in 500 mL PBS to prepare the overnight incubation with the primary antibody against TH (#152, Merck SA, Darmstadt, Germany) made in rabbit in a ratio 1:500. After washing, the secondary antibody (anti-rabbit IgG DAKO #E0432, Agilent, Palo Alto, CA, USA) was applied in the ratio 1:200 overnight. The sections were treated with VECTASTAIN ELITE ABC Peroxidase Vector (Biozol, Eching, Germany) afterwards. DAB Substrate Kit, Peroxidase (with Nickel) from VECTOR was added for 6–10 min each for the optimum result. The stained sections were dehydrated in ethanol and xylol before they were coverslipped.

The 3 µm-sections were hydrated in chronological order using xylol, ethanol, and distilled water. Afterward, the sections were incubated in citrate buffer solution at pH 9 at 90 °C for 40 min, after which they were cooled down to 40 °C for further treatment. The sections were rinsed in a tris-buffer at pH 7.6 and incubated in 3% H_2_O_2_ diluted in distilled water (20 mL H_2_O_2_ ROTIPURAN (Fisher Scientific, Schwerte, Germany) in 180 mL distilled water). The sections were mounted on cover plates and incubated with 200 µL of a solution made of 2 mL Normal Goat Serum in 20 mL tris-buffer and 2 mL Triton solution (SIGMA #SLCD3237). The incubation with the primary antibody overnight at 4 °C and the secondary antibody afterward for 45 min at room temperature was carried out in the same ratio as in the 40 µm-sections. Afterward, Streptavidin-HRP (Southern Biotech, Birmingham, AL, USA) diluted in tris-buffer at 1:1000 was applied for 45 min to continue with the DAB-solution (DAKO) for 7 min each section and Gill’s Hematoxylin II counterstaining. The stained sections were then dehydrated with ethanol and xylol, and the cover slipped.

#### 2.2.5. Luxol Fast Blue Staining

Representative 3 µm-sections were chosen to stain in Luxol Fast Blue (LFB). The paraffin was dissolved in 96% ethanol and incubated overnight at 60 °C in LFB solution (5 mL acetic acid 10% added to 1 g Luxolecht Blau (Fisher Scientific, Schwerte, Germany) solved in 1l of 96% ethanol). Additionally, 0.05% lithium carbonate and 70% ethanol were used to treat the sections further to perform another Periodic Acid-Schiff (PAS) reaction on the sections by adding 1% periodic acid, washing with distilled water afterward, and incubating in Schiff’s reagent. Gill’s Hematoxylin II was used for counterstaining before sequentially dehydrating the stained sections with ethanol (70%, then 96%) and Xylol prior to coverslipping.

#### 2.2.6. Hematoxylin and Eosin (HE) Staining

Further representative 3 µm sections were chosen and treated with xylol, ethanol (96% then 70%), and distilled water before incubating them in Gill’s Hematoxylin II. The sections were then washed and immersed in 05% Eosin until the appropriate staining was achieved. The stained sections were sequentially dehydrated in ethanol (70%, then 96%) and Xylol and coverslipped.

#### 2.2.7. Semi-Quantitative Analysis of DA Cells in VTA Subregions 

**Figure 3:**
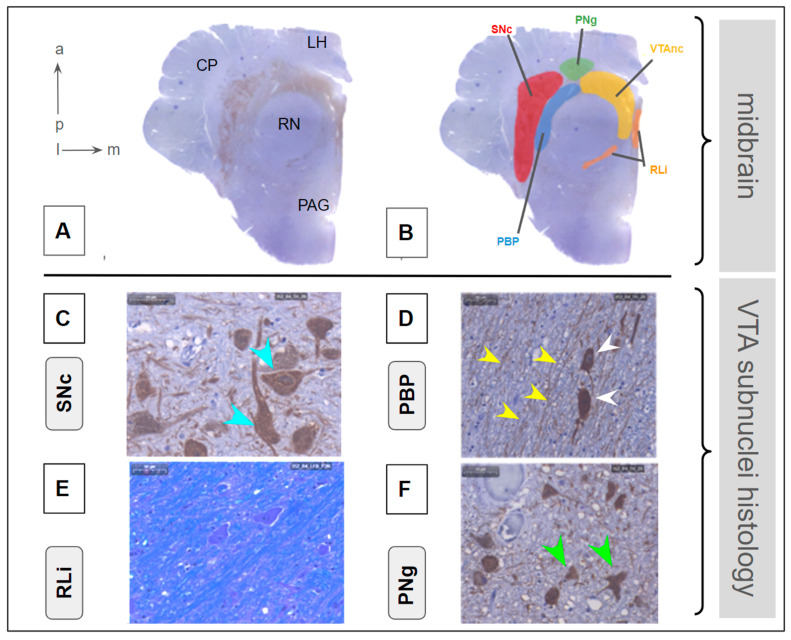
VTA subnuclei—histomorphological characterization and identification in an individual sample. (**A**) identification of a standardized cut in TH staining. (**B**) subnuclei definition and marking according to position and cellular criteria in C-F. C-F, Cellular and fiber characteristics of substantia nigra, pars compacta (SNc), parabrachial pigmented nucleus (PBP), rostral linear nucleus (RLi), and paranigral nucleus (PNg). (**C**) SNc shows the highest density of large DA cell bodies (turquoise arrowhead); (**D**) PBP shows darkly stained cells (white arrowhead). Fibers (yellow arrowhead) are oriented parallel to the superior cerebellar peduncle (not shown); (**E**) RLi is characterized by fine-grained intracellular pigment; (**F**) PNg shows smaller and more densely packed cells than the neighboring SNc. Cells are multipolar (green arrow head). Legend: a, anterior; p, posterior; l, lateral; m, medial; CP, cerebral peduncle; RN, red nucleus; LH, lateral hypothalamus; PAG, periaqueductal gray VTAnc, ventral tegmental area proper nucleus.

After choosing comparable sections that represent the extension of the entire VTA (Figure 3), the subnuclei of the VTA were rendered on the 3 µm TH-stained sections referring to [42] based on A9/A10 discrimination of the DA cells considering their cytoarchitectural features. Afterward, a semi-quantitative analysis was performed on both the density of the TH-positive fibers and the TH-positive cells (cell bodies) on the subnuclei parabrachial pigmented nucleus (PBP), paranigral nucleus (PNg) VTA proper (VTAnc) and rostral linear nucleus (Rli). The substantia nigra pars compacta (SNc) was always used as a relative reference. Cell/fiber densities (reflecting the TH+ immunoreactivity) were estimated and then rated on an ordinal scoring system [43] of four numeral scores (0–3). Densities were rated per visual field with an overview of the subnuclei: 0 = no cells/fibers to 3 = high densities of cells/fibers. As there was a lack of “normal” control samples (S1 one died from an aortic rupture, but tissue quality was not sufficient for analysis), cell density from published work [44] was used as a reference. Subnucleus nomenclature was adjusted as it is inconsistent throughout the literature. The published data do not report depression in the medical background and refer to the respective anatomical correspondence. We agree with [45] that it would be rather preferable to use a common nomenclature. The subnuclei of the sample in the current study were characterized based on a comparison of the cytoarchitecture described in [42].

#### 2.2.8. Statistical Analysis

**Figure 4:**
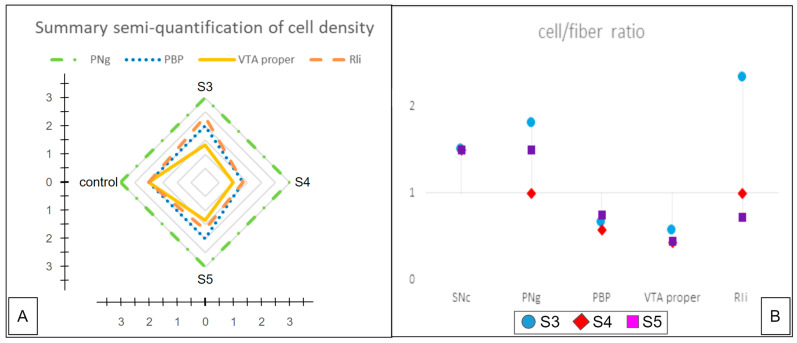
(**A**) summarized data of the semi-quantitative analysis (numerical scores) of cell density. (**B**) cell/fiber ratio (also semi-quantitative analysis). Legend: SNc, substantia nigra pars compacta; PNg, paranigral nucleus; VTA, ventral tegmental area; RLi, rostral linear nucleus. S3, S4, and S5 indicate sample/subject numbers 3–5 (see also Table 1).

Because of the small sample size, statistics are foremost descriptive. Patient demographics and further information are expressed as mean ± standard deviation (SD). Graphs (Figure 4) were created with Windows Excel from Office Professional plus 2016 (Microsoft, USA) and enhanced with Google Slides (Google, USA, 2024 version).

## 3. Results

### 3.1. Autopsy Cases

For this pilot series, seven autopsies were performed between June and October. A specialized forensic pathologist performed the autopsies, reviewed each case, and came to a diagnosis. The mean age of subjects was 58 ± 14.5 years (mean ± standard deviation, SD). There were 3 females. The mean postmortem interval (PMI, interval until autopsy) was 3.6 ± 1.2 (SD) days (86.7 ± 28.3 h). In two cases (S2, S4), suicide was the definite cause of death. Suicide was ruled out in one case (S1) and deemed unclear in another case (S6). The remaining cases were judged as supposable suicides (S3, S5, S7) by the circumstances of the individual case. One case had a proven diagnosis of untreated depression (MDD, case 2). The mean number of hours of monthly daylight in the region of death was 207 ± 30 (SD) hours and was rather similar across all cases. A further characterization of the group, including medical history and autopsy findings, can be found in Table 1.

### 3.2. Histological Tissue Analysis

Following the method development to fine-tune the histological processing of the postmortem human brain tissue, the protocol yielded clear and quantifiable visualizations within the VTA of the target stains, including TH, HE, and LFB (Section 2.2.4, Section 2.2.5, Section 2.2.6 and Section 2.2.7; Figure 3). The different stains allowed the identification of subnuclei and the comparison between the distinct specimens. The 40 µm sections retained the structures better and were used as a validation of the 3 µm sections. The thinner sections were more fragile but revealed more detailed information. As a subnucleus, the paranigral nucleus (PNg, Figure 3) was best identified as it was clearly seen that fibers of the oculomotor nerve traverse the region as previously described [42]. The additional subnuclei were identified by referring to their histomorphological characteristics (Figure 3). The semi-quantitative analysis showed a pronounced DA cell loss in the VTAnc (Figure 4A), whereas the other subnuclei did not reveal any such semi-quantitative change. The comparison of cell density versus fiber density showed clear trends in whether the subnuclei were more likely to express fibers (<1) or cells (>1) (Figure 4B). Only the rostral linear subnucleus showed no clear tendency, which corresponds to the difficulties that occurred while trying to define this subnucleus. The tendency of the VTA proper might have shifted due to the cell loss towards the fibers.

### 3.3. Hybrid Evaluation Including MRI

**Figure 5:**
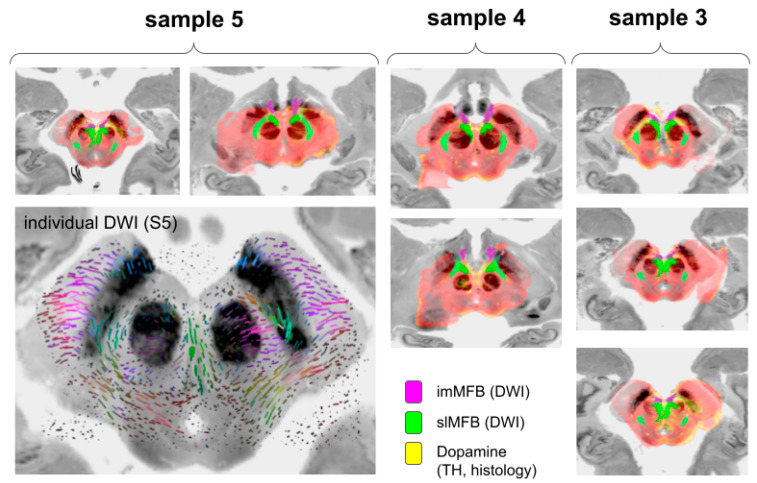
Joint presentation of histology and tractography in a common atlas space (MNI). Samples 3–5. Sample 5, postmortem MRI allowed for individual DWI imaging. Streamline renditions show color-coded fiber directions. Legend: imMFB, inferomedial branch of the medial forebrain bundle (MFB); slMFB, superolateral MFB; TH, tyrosine hydroxylase.

**Figure 6:**
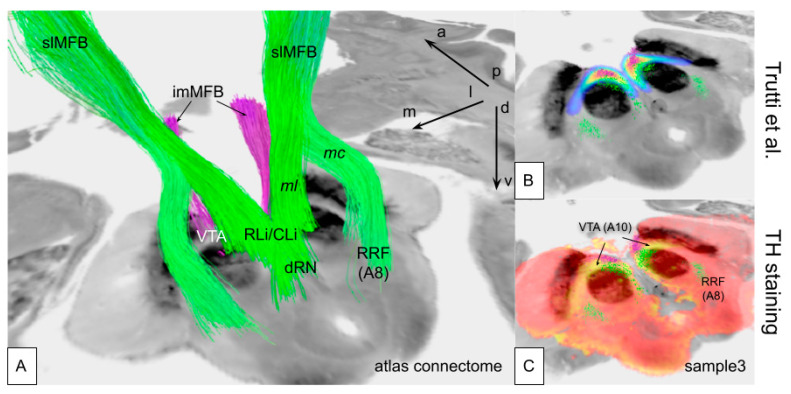
Three-dimensional depiction of histology and fiber tract anatomy in MNI space. (**A**) differentiation between the route of imMFB and slMFB. (**B**) same region depicted with a probabilistic atlas overlay from [46]. (**C**) histological specimen from this study (sample 3) integrating DA (yellow) and in-plane fiber tract depiction (imMFB, purple; slMFB, green). Legend: slMFB, superolateral branch of the medial forebrain bundle; imMFB, inferomedial branch of medial forebrain bundle; ml, mesolimbic portion; mc, mesocortical portion; RLi, rostral linear nucleus; CLi, caudal linear nucleus; dRN, dorsal raphe nucleus; RRF, retro-rubral field (A8); VTA, ventral tegmental area (A10).

Using the postmortem MRI as a scaffold, the hybrid presentation of individual TH histology and diffusion-weighted imaging (DWI), as well as atlas data, was feasible (Figure 5 and Figure 6). A group analysis of individual fibers based on DWI MRI studies from specimens was not performed since not all MRI scans showed sufficient DWI quality. The imMFB and slMFB fibers from an MNI-based atlas [13] were correlated with individual histological samples from (S1, S2, S3; Figure 5). Furthermore, the slMFB midbrain fiber receiving regions and imMFB sending regions could spatially be co-localized with individual histologically determined VTA regions in our three samples (Figure 5) and showed concordance with available penetration patterns. A comparison to a recent probabilistic VTA atlas of Trutti et al. [46] showed a comparable A10 region extension in the midbrain (Figure 6).

## 4. Discussion

**Figure 7:**
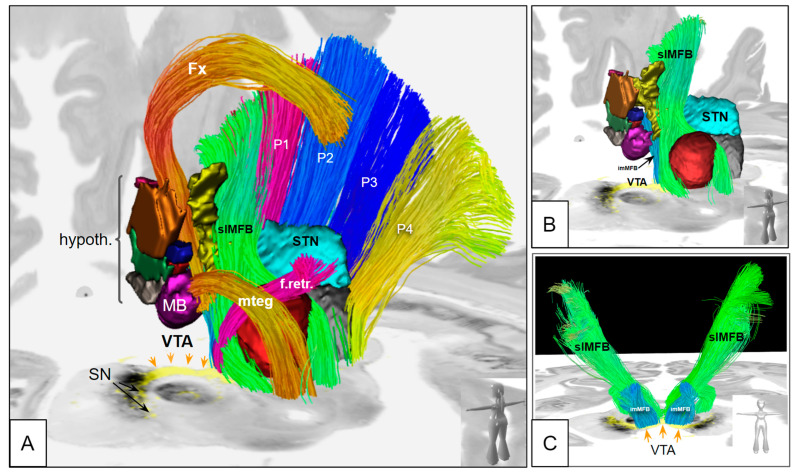
Atlas integrations in MNI space and conjoined histology. (**A**) full anatomy with fiber atlas data and hypothalamus data from [78]. (**B**) reduced version showing slMFB’s principle route into the midbrain and imMFB out of the midbrain. (**C**) view from anterior. Legend: slMFB, superolateral branch of the medial forebrain bundle; imMFB, inferomedial branch of medial forebrain bundle; Fx, fornix; VTA, ventral tegmental area; MB, mammillary body; hypoth, hypothalamus; SN, substantia nigra; STN, subthalamic nucleus; mteg, mammillo-tegmental tract; f.retr., fasciculus retroflexus (of Meynert); P1, Arnold’s bundle; P2, corticobulbar tract; P3, corticospinal tract; P4, parieto-temporo-occipital tract.

The main aim of this proof-of-concept study was to define a workflow, allowing the generation of a general but at the same time detailed histological overview of the human midbrain VTA and its dopaminergic pattern and connectivity in subjects who have committed suicide in comparison to normal control subjects who died of non-suicide causes and to extrapolate this information to MDD. We have additionally found some very early and preliminary hints for a locally changed DA cell/fiber distribution in the VTA that might potentially serve as an indicator for suicide behavior/MDD in the future. An implementation also with different atlas information in the MNI space appears feasible (Figure 7).

### 4.1. Dopamine in the VTA as a Biomarker for MDD and Its Potential Link to the Risk of Suicide

Subcortical regions like the VTA (Dopamine), the locus coeruleus (LC, Noradrenaline), and the dorsal raphe nucleus (DRN, serotonin) are the physiological origin of transmitter systems addressed with antidepressant medication [4,47]. Mayberg et al. discussed the role of cortical circuitry in moderating subcortical emotion-related (limbic) regions already in their early work [48] and recently, the role of a downregulation of the DA system by an overactive infralimbic cortex (the rodent analogon to the evolutionary more developed SCG) has been highlighted [49]. It is of note, however, that the mere substitution of oral DA or its analogs is not typically sufficient to treat major depression, pointing to the role of phasically acting DA systems in mood and behavior regulation [49]. The VTA—addressed here—has a special role as a regulator of the mesolimbic/mesocortical DA system. It is the seat of the A10 group of DA neurons. The VTA is the midbrain origin of the medial forebrain bundle (mfb), which is a complex projection pathway accommodating all the above-mentioned and more transmitters on the way to the basal forebrain and the prefrontal cortex [50,51]. The slMFB (in primates) is supposed to serve mainly as a glutamatergic feedback loop from the prefrontal cortex [52]. Rodent studies, which we do not address in further detail here, underpin the role of DA in depression [21,22,23,24,53,54,55,56]. It is of note that in rodents, a depressive phenotype is in part related to reduced DA transmission along the VTA-PFC axis. With respect to the role of the VTA in MDD, Conway and coworkers have shown in their clinical vagus nerve stimulation work that the VTA, among other regions, showed higher metabolism at the 12-month interval in responders [57]. Recently, an aggregation analysis of lesions for depression in multiple sclerosis also pointed to the ventral midbrain and the VTA as a critical region [58].

One of the other and potentially more prominent subcortical transmitters in the context of MDD and suicide is serotonin (5-HT) [59]. While we will not go into too much detail here, it should be mentioned that 5-HT plays an important role in the discussion on depression and suicide behavior genesis. The discussion is fueled by the widespread use of SSRI [60] as one important drug in depression treatment. The VTA is in its midline nuclei (RLi/CLi) connected to the dorsal raphe nuclei [42] as the main seat of 5-HT cells, which project out of the brainstem via the MFB [61]. It might in the future also be interesting to look at this midline repository of 5-HT cells within our described research framework. For a more recent discussion on the role of 5-HT, please refer to [62].

In search of the origin of MDD, numerous other mechanisms have been proposed [4]. Recently, the literature has focused on the ventromedial prefrontal cortex (vmPFC) and, here specifically, on an overactivity in the subgenual cingulate gyrus (SCG, cg25). Mayberg et al. have highlighted the specific role of SCG activity in depression and sadness [48]. This work already mentioned the reciprocity of SCG to parts of the dorsolateral prefrontal cortex (dlPFC). DBS of the SCG region itself appears to be an effective treatment of very treatment-resistant MDD [63] if used in combination with tractographic targeting technologies [64]. Tractographic analyses of slMFB DBS have shown that there is a clear overlap between the slMFB and SCG circuitry. Moreover, frontal cortex white matter architecture seems to be related to the outcome of DBS in TRD close to the VTA (slMFB DBS) [65]. Currently, enhanced treatment protocols of noninvasive electrical stimulation using repetitive transcranial magnetic stimulation (rTMS) focus on individual and functional investigations regarding the SCG and dlPFC connectivity [66]. The resting state functional magnetic resonance imaging derived inverse correlation of SCG and dlPFC is currently researched on an individual basis and sought to enhance rTMS efficacy [67].

### 4.2. Choice of Midbrain Specimens

Regarding the medical background of our midbrain samples, a direct link to depressive behavior cannot be inferred since suicide is not necessarily a result of depression [68]. Some studies link a reduced DA turnover to MDD suicides [69], while other work does not describe the involvement of D1/D2 receptors [70]. Fitzgerald and coworkers describe a disbalance between DA transporter and DA receptors in suicide behavior [71]. Based on clinical and animal experimental considerations, it appears to be promising to approach the problem of transmitter changes in MDD via tissue from suicide victims and—according to the reasoning above—with a detailed look at their brainstems. It is not clear in this respect if patients committing suicide might show a special DA anatomy and metabolism [72] (see also Section 4.6).

### 4.3. Postmortem Interval

Because of the prospective nature of this work, the PMI was 3.6 days (86.5 h) which per se is a rather long time for brain tissue characterization. At the same time, the cooling conditions of the corpses in such a setting cannot entirely be controlled, depending on the time between suicide and the finding of the body, environmental factors like room temperature, and the cooling process during the transfer of the body to forensic medicine. However, the TH staining—as a protein staining used here—appears to be rather robust with respect to these effects [73], as long as the tissue has, in principle, not undergone a too severe postmortem decomposition. We have, in the low number of tissue samples in this work so far, not observed obvious detrimental effects. It is, however, clear that earlier autopsies in the range of up to 36 h are preferable (our samples S2, S3, and S7 are closest with 55–60 h PMI). Work in rodents has shown that, in principle, rather stable conditions for DA receptor staining can be maintained up to 24 h post mortem [74]. However, given these constraints, it will be challenging to develop more differentiated evaluation protocols like autoradiography or mRNA for the tissue samples acquired in this workflow. With the expansion of our tissue bank, we will address in future work what effect PMI has with respect to our DA analyses. Please also refer to the limitations (Section 4.6).

### 4.4. Differential DA Cell Changes in Subnuclei of the VTA

In previous work on MPTP-lesioned monkeys, a loss of the overall DA transmission in the VTA-nucleus accumbens pathway has been shown to result in apathetic behavior [20]. This work did not focus on any subnucleus of the VTA. In rodents, the PBP has been shown to have the strongest connection to the nucleus accumbens and is thus expected to play a major role in the reward system in rodents [75]. It might, therefore, be considered to be surprising that the VTA proper in our work showed a much clearer DA cell loss than the PBP. However, Trutti et al. [45] have discussed the ambiguities of the naming of the VTA proper nucleus (VTAnc). In their view, the VTAnc is intercalated between the paranigral and PBP subnuclei, building the analog to what is sometimes called the “lateral VTA”. Our recent work in the marmoset monkey clearly found termination of PFC fibers in the PBP nucleus [52], which is—as part of the lateral VTA—the target region of slMFB DBS in MDD and OCD [11,76,77], also referred to as the “VTA terminal field” [13]. Figure 5 and Figure 6 show how the lateral VTA serves as the fiber receiving region for the slMFB and additionally as the exit region for the imMFB. Because of the nomenclature ambiguities regarding the VTAnc and the small number of samples in this work, one might rather speak of a reduction of DA cells in “the lateral VTA” as a potential and soft finding. Future work on this project might shed more light on exactly this problem.

### 4.5. Seasonal Photo-Period and DA Cells

There is recent literature pointing at differences in DA cell count with respect to sunlight exposure in individual subjects [33,34]. We have therefore calculated the assumed exposure to sunlight in the region where death occurred (Table 1). In an expanded sample size, a seasonal balance must be achieved to control for the influence of sunlight on the DA cell count in the distinct VTA regions. In our sample here (especially S3–S5), the seasonal photo-period was uniform and is not regarded as having influenced our results. We can, of course, not be entirely sure about the true sunlight exposure, which is related to individual behavior.

### 4.6. Limitations

Acquiring tissue samples from MDD in a prospective fashion is notoriously difficult. Due to a lack of access to a brain bank, we have collected tissue samples aiming to include potential suicide victims and non-suicide controls. A certain overlap of suicide with MDD can be suspected. It is, moreover, thinkable that only a certain phenotypical subclass of MDD patients, in fact, commit suicide [79]. In this context, the literature describes “impaired decision-making” as a typical vulnerability for suicide [79,80]. Intact decision-making is subcortically directly linked to DA [81] and likely to the functioning of the slMFB [82].

Other diseases like schizophrenia have been related to alterations in the DA system [83], so it will be crucial to disentangle these subjects from purely depressed ones in future analyses. Suicide in itself is a complicated matter [68,79,84]. We will not discuss this in detail problem, but it is clear that upon suspected suicide, a thorough post hoc workup of medical history, including interviewing of relatives and treating physicians, is necessary to confirm MDD (as the main cause) and other causes. The main limitation of this work is the number of tissue samples, which is too low to draw further conclusions on DA anatomy. Thus, DA related results reported here must be regarded as preliminary and exploratory at best since our main intention is to describe the intricate pipeline necessary for such research. We will address this with a growing sample number in future work.

TH staining is a rather nonspecific marker and is not able to differentiate between any catecholamine cell population (noradrenaline, NA; norepinephrine, NE and Dopamine, DA) [85]. Accordingly, Mejias-Aponte and coworkers have shown a widespread innervation of A10 and A8 cell groups by NA and NE projection from various regions [86]. In this respect, our detection of midbrain TH+ cells might be regarded as biased. However, NA and NE cell bodies are not typically found as part of the A8-A10 cell groups, and therefore, the localization of TH+ stained DA cell bodies in the typical location of the VTA appears valid [61,87]. With respect to the fiber count, we potentially will not differentiate (projecting) fibers from any TH+ cell group in our target region, making this analysis less reliable. We will, in the future, refine our analysis and use DA receptor stains to further differentiate cell populations.

The PMI is rather long, and this fact might potentially outbalance the availability of precious human tissue samples. Since we have so far predominantly sampled suspected suicide cases for this pilot, there is no real control group in our series. In the future, we plan to additionally collect tissue from subjects who have died of other non-suicide causes (like sudden death from heart diseases). Factors like the cooling condition of the corpse, the age and the cause of and the rapidity of death may lead to a difference in the behavior of the tissue itself during processing [70], which ideally should be controlled throughout the whole procedure. We have so far not looked at DA receptor polymorphisms but will look in the future for feasibility regarding the rather long PMI of our samples.

In the future we will use stereoscopic evaluation of cell counts, a technique which was not available for this work. Other factors, such as substance abuse disorders (alcohol, drugs) and other diseases associated with dopaminergic lesions, such as Parkinsonism and Schizophrenia, must be clearly excluded, as well as neuroleptic medication, which could influence DA anatomy [88].

## 5. Conclusions

This study represents our first attempt to investigate potential dopaminergic cell/fiber alterations and to correlate these with suicide (and MDD) using human autopsy midbrain specimens. The principal idea is to investigate a potential connection between DA transmission and MDD. The idea to investigate DA nuclei in the brain stem is not new and has been previously reported. However, as a refined methodology, we have more specifically investigated DA midbrain regions, which have previously been related to reward and decision-making. We have included investigations of specific subnuclei of the ventral tegmental area (VTA, A10), a region that might play a role in DBS for MDD. Moreover, we have created and here described an investigation pipeline to further scrutinize such regions and to compare them in a common space (MNI), conjoined with fiber tract anatomy to allow for group-level analyses. Future work should focus on an agreed nomenclature for the subnuclei of the human VTA. An increased subject sample number in this growing tissue database within the framework of an ethical protocol—including reaching out to closest relatives to clear up circumstances of death and medical conditions, leading to a better sample characterization in every case—is the focus of our current research.

## Figures and Tables

**Table 1:** Specimens and characteristics.

No.	Age [Years]	Gender	PMI [Hours]	Month of Death	Hours of Monthly Sunlight *	Cause of Death	Suicide ?	MD ?	Medical History and Autopsy Findings	Workup
S1	82	f	120	June	240	hemorrhagic shock after aortic rupture	no	no	aortic valve replacement, hypertrophic heart disease, myocardial fibrosis	fixation, histological workup, and staining
S2	67	m	55	July	260	self-inflicted stab and cutting injuries	yes	yes	untreated depression	fixation, histological workup, and staining
S3	55	m	60	Sept.	190	CO-intoxication (inhalation)	supposable	no	massive hypertrophic heart disease, coronary heart disease, kidney cysts, cerebellar subarachnoid cyst	fixation, MRI, full histological workup
S4	62	m	96	Oct.	190	drug intoxication	yes	unknown	hypertrophic heart disease, pulmonary emphysema, renal cyst right, prostate hypertrophy	fixation, MRI, full histological workup
S5	36	m	120	Oct.	190	mixed intoxication (alcohol, Pregabalin, Promethazine)	supposable	no	abuse of alcohol, medication, and drugs; steatosis hepatis	fixation, MRI, full histological workup
S6	58	f	96	Sept.	190	cardiac arrest (arrhythmogenic)	unclear	unknown	suicide attempt 11 days before death; hypertrophic heart disease, myocardial fibrosis, pulmonary emphysema, steatosis hepatis	-
S7	48	f	60	Sept.	190	CO-intoxication	supposable	no	mitral valve regurgitation, uterine myomas	fixation, MRI

Legend: S1–S7, specimens 1–7; f, female; m, male; no., number * daily sunlight average for the region of Freiburg, Germany. (https://weather-and-climate.com/average-monthly-hours-Sunshine,freiburg-im-breisgau,Germany, accessed on 1 May 2024).

## Data Availability

Data will be made available upon reasonable request to the corresponding author. The data are not publicly available, because of ethical concerns.

## Abbreviations

5-HT	Serotonine
DA	Dopamine
DBS	Deep Brain Stimulation
dlPFC	dorsolateral prefrontal cortex
dmPFC	dorsomedial prefrontal cortex
dRN	dorsal raphe nucleus;
imMFB	inferomedial branch of the medial forebrain bundle (primate);
MDD	major depressive disorder
mfb	medial forebrain bundle (rodent)
NAC	nucleus accumbens septi
VTA (A10)	ventral tegmental area (of Tsai):
RLi	rostral linear nucleus;
CLi	caudal linear nucleus;
PN	paranigral nucleus;
PBP	parabrachial pigmented nucleus;
VTAnc	ventral tegmental area proper nucleus;
rrf	retro-rubral field (A8);
SCG	subgenual cingulate gyrus (cg25)
slMFB	superolateral branch of the medial forebrain bundle (primate);
SNr	substantia nigra pars reticulata;
SNc (A9)	substantia nigra pars compacta;
SSRI	selective serotonin reuptake inhibitors
STN	subthalamic nucleus;
TH	tyrosine hydroxylase
TRD	treatment-resistant depression
vmPFC	ventromedial prefrontal cortex
